# Parents’ Perspectives on Counseling for Fetal Heart Disease: What Matters Most?

**DOI:** 10.3390/jcm11010278

**Published:** 2022-01-05

**Authors:** Alexander Kovacevic, Annette Wacker-Gussmann, Stefan Bär, Michael Elsässer, Aida Mohammadi Motlagh, Eva Ostermayer, Renate Oberhoffer-Fritz, Peter Ewert, Matthias Gorenflo, Sebastian Starystach

**Affiliations:** 1 Department of Pediatric and Congenital Cardiology, Heidelberg University Hospital, 69120 Heidelberg, Germany; Matthias.Gorenflo@med.uni-heidelberg.de; 2 Institute of Preventive Pediatrics, Faculty of Sport and Health Sciences, Technical University of Munich, 80992 Munich, Germany; annette.wacker-gussmann@tum.de (A.W.-G.); aida_motlagh@yahoo.de (A.M.M.); renate.oberhoffer@tum.de (R.O.-F.); 3 German Heart Center Munich, Department of Pediatric Cardiology and Congenital Heart Defects, 80636 Munich, Germany; ewert@dhm.mhn.de; 4 Max Weber Institute for Sociology, Ruprecht Karls University Heidelberg, 69115 Heidelberg, Germany; stefan.baer@mwi.uni-heidelberg.de; 5 Department of Gynecology and Obstetrics, Heidelberg University Hospital, 69120 Heidelberg, Germany; Michael.Elsaesser@med.uni-heidelberg.de; 6 Department of Obstetrics and Gynecology, Klinikum Rechts der Isar, Technical University of Munich, 81675 Munich, Germany; eva.ostermayer@t-online.de; 7 Institute of Medical Sociology and Rehabilitation Science, Charité University Medicine Berlin,10117 Berlin, Germany; sebastian.starystach@charite.de

**Keywords:** fetal cardiology, parental counseling, social science, parental needs

## Abstract

After diagnosis of congenital heart disease (CHD) in the fetus, effective counseling is considered mandatory. We sought to investigate which factors, including parental social variables, significantly affect counseling outcome. A total of *n* = 226 parents were recruited prospectively from four national tertiary medical care centers. A validated questionnaire was used to measure counseling success and the effects of modifiers. Multiple linear regression was used to assess the data. Parental perception of interpersonal support by the physician (β = 0.616 ***, *p* = 0.000), counseling in easy-to-understand terms (β = 0.249 ***, *p* = 0.000), and a short period of time between suspicion of fetal CHD, seeing a specialist and subsequent counseling (β = 0.135 **, *p* = 0.006) significantly improve “overall counseling success”. Additional modifiers (e.g., parental native language and age) influence certain subdimensions of counseling such as “trust in medical staff” (language effect: β = 0.131 *, *p* = 0.011) or “perceived situational control” (age effect: β = 0.166 *, *p* = 0.010). This study identifies independent factors that significantly affect counseling outcome overall and its subdimensions. In combination with existing recommendations our findings may contribute to more effective parental counseling. We further conclude that implementing communication skills training for specialists should be considered essential.

## 1. Introduction

Congenital heart disease (CHD) affects approximately 9 per 1000 newborns [1]. Technical advances and improvements in national fetal anomaly screening programs have led to higher prenatal detection rates over the past decades. For duct-dependent cardiac lesions prenatal diagnosis has the potential to improve postnatal morbidity and mortality [2,3,4,5]. Standards for effective parental guidance in this context have been proposed [6,7,8]. Nevertheless, parents’ needs are not always met, and there are few studies aiming to identify the most effective counseling techniques [6,9,10,11].

Receiving the message that the unborn child has a heart defect carries the risk of maternal, but also paternal psychological distress, increased levels of anxiety, and depression. There is an association between maternal stress and preterm delivery, small for gestational age newborns, and late sequelae in children, such as alterations in somatic and neurocognitive developments [12,13,14,15,16,17,18,19,20].

Continuing research in this field to present evidence-based guidance for professionals is therefore warranted. In earlier research, our group demonstrated that the structure of the counseling setting, e.g., by using a separate counseling room or providing additional written information and illustrations on the diagnosed fetal heart disease, influences “overall counseling success”, and its subdimensions such as “transfer of medical knowledge” or “transparency regarding the treatment process” [21,22].

This study aims to develop a more differentiated understanding with the help of multiple linear regression models. Besides the structure of the counseling situation and contextual factors (e.g., counseling during the COVID-19 pandemic) as independent variables, we controlled for individual characteristics of the parents since the respective parent plays a significant role in the counseling situation. It is well described that social factors, such as educational levels, social status or gender, may significantly influence the interaction with healthcare specialists [23,24]. In the specific situation after a diagnosis of CHD in the unborn child, these factors may affect the parents’ understanding of the diagnosed fetal heart disease, treatment options, aspects of the child’s long-term outcome, and their own coping mechanisms.

Therefore, the objective of this study was to investigate which factors (structure of the counseling situation, contextual factors, socio-demographics) play an independent and significant role in determining the outcome of parental counseling for fetal heart disease.

## 2. Materials and Methods

In this multicenter study, parents were recruited from November 2016 to December 2020 from four tertiary medical care centers at two locations (location A: centers 1 and 2; location B: centers 3 and 4). Beforehand, database analyses were performed to identify children with a prenatal diagnosis of CHD (diagnosed during fetal anomaly screening or after suspicion of fetal CHD raised by referring centers or obstetricians). Accordingly, this is a quantitative study with retrospective and prospective data acquisition. Descriptive statistics were used to determine the sample structure (Appendix A, Table A1).

A questionnaire was designed with appropriate queries grouped into five subdimensions of counseling success: (1) “Transfer of Medical Knowledge”, (2) “Transparency Regarding the Treatment Process”, (3) “Trust in Medical Staff”, (4) “Perceived Situational Control”, and (5) “Coping Resources” (Table 1). The five subdimensions are based on a systematic literature review and analyses of daily practice experiences [25]. Corresponding scales to measure these dimensions were constructed and successfully tested for internal consistency. The Likert items measure parental agreement or disagreement in accordance with each query from the questionnaire on a five-point scale: strongly agree, agree, partially agree, disagree, and strongly disagree (for statistics each point scale is converted into a number from one to five: 1 = strongly agree, 2 = agree, 3 = partially agree, 4 = disagree, and 5 = strongly disagree). To determine “overall counseling success” a sum-score of 16 Likert-items (items = queries from the questionnaire) was constructed. The sum score has a possible range from 16 = completely unsuccessful to 80 = completely successful. The sum score “overall counseling success” shows good internal consistency with a Cronbach’s α coefficient of 0.904. In the same manner, the sum-scores for the subdimensions of counseling success were successfully constructed, using the items of the respective subdimension (Table 1).

Furthermore, the questionnaire consists of 5-point-Likert-scale items assessing the structure of the counseling situation, e.g., “Was the counseling session frequently interrupted by the physician?”, as well as questions concerning socio-demographic factors of the counseled parent [25].

Parents were interviewed by two medical students during routine follow-up visits of their children in the participating Pediatric Heart Centers, who were not involved in diagnostic procedures or parental counseling to avoid a response bias. Alternatively, questionnaires were issued during the visits or were sent to the families. Contextual and meta information such as the location of the treatment facility were gathered and incorporated into the resulting dataset. Data from questionnaires were excluded if parental counseling had been performed via interpreters.

Multiple linear regression models were used to identify significant and independent influential factors on the dependent variable “overall counseling success” and its five subdimensions. The independent variables entailed social, spatiotemporal, informational (see Table 1) and contextual factors, e.g., location, counseling during the COVID-19-pandemic, and control variables such as parental age or gender (see Appendix A, Table A1, for a full list). The sum-scores of “overall counseling success” and the sum-scores of the respective subdimension of counseling success were used as dependent variables.

Statistical analysis was performed using IBM^®^ SPSS^®^ Statistics Version 26 (IBM Germany, Ehningen).

The study’s protocol was approved by the local Ethics Committees of each involved Medical Faculty and participants provided their written informed consent before their inclusion in the study, which was conducted in accordance with the Declaration of Helsinki.

## 3. Results

### 3.1. Sample Structure and Counseling Success

From November 2016 until December 2020 *n* = 226 individuals were recruited from four national tertiary medical care centers (for detailed sample structure see Appendix A, Table A1). The drop-out rate was 57%.

In the participating centers, three fetal cardiologists and three maternal-fetal medicine (MFM) specialists were involved in fetal scanning and parental counseling. Counseling was performed either by cardiologists or MFM specialists separately or combined, depending on the location of referral.

At the time of counseling, the parental mean age was 34.6 years (SD 5.4); 59.3% of parents were female and 40.7% male.

Overall, 90.3% of couples were in a permanent relationship, and 9.7% were not.

Parental educational level according to ISCED (International Standard Classification of Education) [27] was high in 55.6%, medium in 38.2%, and low in 6.2%.

Parental social status according to occupation was high in 29%, medium in 43.3%, and low in 27.6%.

The native language was German (=language of counseling) in 83.2%, and 16.8% of parents had another native language.

Preexisting medical knowledge was held by 31.1% vs. 68.9% without preexisting expertise.

Median gestational age when fetal cardiac diagnosis was made was 23 weeks (range 9–38 weeks).

Diagnosed fetal CHD was classified as high-risk in 57.1%, medium risk in 31.3%, and low risk in 11.6% [7]. Table A2 (Appendix A) summarizes fetal cardiac and extra-cardiac anomalies.

All pregnancies ended in live births (*n* = 4 were lost to follow-up).

Overall counseling success was high, and considered to be successful in 47.5%, satisfying in 52%, and unsuccessful in 0.5% (as assessed by sum-scores; for counseling success in the analytical subdimensions see Appendix A, Table A3).

### 3.2. Multiple Linear Regression

The multiple linear regression models explain, between a moderate to a high degree, the variances of the dependent variables of counseling success overall and in its subdimensions, with the exception of perceived situational control (corrected R^2^ = 0.141).

#### 3.2.1. Overall Counseling Success

In the multivariate analysis, four of the independent variables significantly influence “overall counseling success” (Table 2):

1. “Little time was lost between a potential cardiac diagnosis and the proper counseling after making the correct diagnosis by a specialist” (temporal aspect); β = 0.135 **, *p* = 0.006.
2. “During the conversation, the topic was explained to me in an easy and understandable way (for example, without technical terms or phrases)” (social aspect); β = 0.249 ***, *p* = 0.000.
3. “I experienced strong support from the physician who conducted the conversation” (social aspect); β = 0.616 ***, *p* = 0.000.
4. The location of the treatment center β = 0.102 *, *p* = 0.037.

#### 3.2.2. Effects on Subdimensions

##### Social Factors

Parental experience of strong interpersonal support by the physician positively influences counseling success in all five subdimensions ((1). transfer of medical knowledge, *p* = 0.000; (2). transparency regarding the treatment process, *p* = 0.000; (3). trust in medical staff, *p* = 0.000; (4). perceived situational control, *p* = 0.000; and (5). coping resources, *p* = 0.000). Frequent interruptions of the counseling session negatively influence counseling success in the subdimension “transparency regarding the treatment process” (*p* = 0.000). Counseling in easy-to-understand terms positively influences counseling success for “transfer of medical knowledge” (*p* = 0.000), “coping resources” (*p* = 0.003) and “transparency regarding the treatment process” (*p* = 0.038). Non-significant results are shown in Table 2.

##### Spatiotemporal Factors

A short period of time between suspected fetal heart disease, making the correct diagnosis and counseling by a specialist positively influences “coping resources” (*p* = 0.004) and “transparency regarding the treatment process” (*p* = 0.010). A lack of an appropriate or separate counseling room negatively influences “trust in medical staff” (*p* = 0.021). Non-significant results are shown in Table 2.

##### Informational Factors

An unfulfilled parental need to receive more information on fetal heart disease negatively influences “transfer of medical knowledge” (*p* = 0.000), whereas providing additional information positively influences counseling success for “transparency regarding the treatment process” (*p* = 0.010). Non-significant results are shown in Table 2.

##### Control Variables

Parental age, i.e., older age, positively influences “perceived situational control” (*p* = 0.010). If parental first language corresponds to the language in which counseling was conducted (German) “trust in medical staff” (*p* = 0.011) is positively influenced. The severity of fetal CHD positively influences “coping resources” (*p* = 0.011). Counseling success according to location of the treatment center differs for “trust in medical staff” (*p* = 0.028) and “transparency regarding the treatment process” (*p* = 0.022). Non-significant results are shown in Table 2.

## 4. Discussion

After making a diagnosis of CHD in the fetus, effective parental counseling is considered mandatory. Standards for counseling have been proposed, but parental needs often differ from specialists’ expectations. It is well described that the patients’ social factors, such as educational levels, social status or gender, may affect interaction with healthcare specialists due to differing competences in understanding and applying health information [23,24]. Therefore, we also measured the influence of parental social variables to control for their independent effects.

Interestingly, we cannot confirm that factors such as parental educational levels, social and relationship status, or gender significantly influence overall counseling success or any of its subdimensions. This may be explained by the nature of the situation. Since the diagnosis of a CHD in the unborn child is a traumatic event for parents, their individual characteristics may not be converted effectively into coping mechanisms. Factors that moderate the parents-physician interaction are therefore expected to play a significant role, which is demonstrated by our findings. The results of our model point to the importance of interpersonal skills of the physician. The specialist’s ability to support parents is a main factor to ensure effective counseling. In addition, knowledge and skills on how to explain particular complex cardiac diagnoses and postnatal therapeutic options, including the child’s long-term outcome, by using understandable terms, further affects counseling outcome. Sufficient interpersonal and communication skills cannot be taken for granted, and should therefore be considered as essential in the training for physicians working in this field.

We can further demonstrate that the temporal aspect of the process plays a central role. We found that “fast processing” is a main factor to achieve parental overall counseling success. Accordingly, in case of a potential CHD in the fetus, waiting time for appointments with specialists should be as short as possible.

Additional modifiers influence subdimensions of counseling, such as the availability of a designated counseling room or additional informational material on diagnosed CHD (illustrations or online links with adequate data). These effects were described by our group earlier, as well as the relatively low counseling success for “perceived situational control” (Appendix A, Table A3) [21,22]. Exactly how counseling influences perceptions of situational control should be the subject of further research. It must be assumed that this dimension is not yet properly addressed in the current counseling situation, or can hardly be addressed, since situational control does not only refer to the acute situation, but possibly to the entire life span. This may correspond to our finding that with increasing parental age, counseling success for “perceived situational control” is higher (Table 2). For concrete practice, the recommendation for early psychological care and support by social workers or cardiac nurse specialists can be derived, and may potentially be important for expecting parents at younger age.

“Trust in medical staff” is influenced by the parents’ native language. If it differs from the language counseling is conducted in, early inclusion of interpreters may be crucial for effective counseling concerning this subdimension. This is in line with our previous findings [21,22]. It is of note that for this sample, parental language skills did seem adequate during the original counseling sessions and interviews, otherwise these data would have been excluded. Furthermore, data on counseling via interpreters have not been included as the content and quality of translations would not have been assessable for the purpose of this study. Against this background, the effect of the parents` native language could also point to cultural differences in terms of the way the hospital organization and the medical professional is perceived in relation to their trustworthiness. Therefore, it appears worthwhile to address parental cultural and ethnic backgrounds in a future study.

However, there are also some unexpected results. Locational effects on “overall counseling success” cannot be explained sufficiently as structures are very similar in the participating tertiary medical care centers, and only qualified consultants with at least 15 years of working experience in this specialty were involved in fetal imaging and subsequent parental counseling in this sample. Still, differing levels of experience in counseling cannot be ruled out that may have influenced our findings.

If complex fetal CHD was diagnosed, counseling success for parental “coping” was found to be positively influenced, which is not in line with our previous data [21,22]. We assume that the COVID-19 pandemic may have influenced our current results as, during the pandemic, routine day-to-day operations were temporarily reduced, thus leading to a more stable counseling setting with less interruptions and time pressure. However, COVID-19 does not influence counseling success, which is in line with our previous study on the impact of the still ongoing pandemic on fetal cardiac care [28].

**Figure 1:**
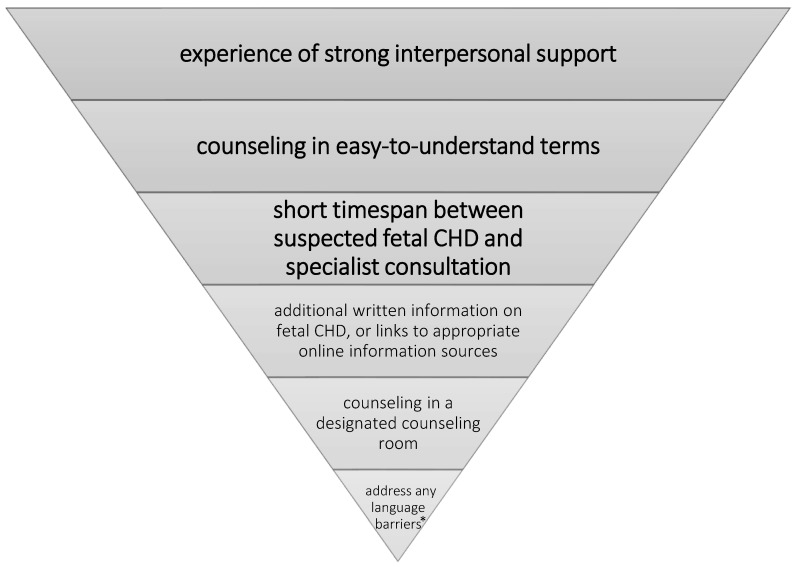
Proposed hierarchy of parental needs contributing to “overall counseling success”, and for its subdimensions displayed as inverted pyramid (based on multiple linear regression models); * i.e., even subtle parental language barriers.

Figure 1 shows a weighting of factors displayed as inverted pyramid contributing to “overall counseling success” and for its subdimensions, thus displaying a proposed hierarchy of parental needs for counseling in this context, based on our current data.

In summary, our results may serve as paradigm to develop strategies to optimize fetal cardiac care in terms of improving the effectiveness of parental counseling [21,22]. Furthermore, as national guidelines are scarce, our results may be valuable to propose evidence-based strategies and suggest topics to be included during conversation [29]. In addition, communication skills training for “breaking bad news” in Fetal Cardiology should be implemented. In contrast to other medical fields, such as oncology or neurology, this subject seems underrepresented for this specialty [30,31].

### Limitations

Parents’ cultural or ethnic backgrounds have not been assessed and may have influenced the results. However, we could demonstrate an effect of parental native language on “trust in medical staff”, which we believe may correspond to potential effects of culture or ethnicity. Still, generalizability of our findings may therefore be impaired.

The effect of the location on counseling success may be due to differences in the consulting style of individual physicians, and therefore serves as moderating factor, which cannot be understood any further with the methods applied. However, this fact points towards a need for further research.

## 5. Conclusions

This study identifies independent factors that significantly affect counseling outcome overall and its subdimensions. A short time span to see a specialist, counseling using understandable terms and experience of strong interpersonal support by the physician during the conversation are valued most by parents. By implementing defined structures in fetal cardiology programs, including communication skills training for specialists, effective parental counseling after fetal diagnosis of CHD is more likely to be achieved. Our methodical approach may serve as a model to optimize existing fetal cardiology programs, select topics for training of specialists, and finally be valuable to propose national evidence-based guidelines.

## Figures and Tables

**Table 1:** * Subdimensions (1–5) of counseling from the Likert scale questionnaire (parents answered on a five-point scale: strongly agree, agree, partially agree, disagree, and strongly disagree) with the corresponding queries (= items). α = Cronbach’s α coefficient, i.e., a reliability coefficient with a range from 0 to 1.0; values > 0.7 show good, > 0.8 very good, and > 0.9 excellent internal consistency [25,26].

**1. Transfer of Medical Knowledge** (sum-score range = 5 to 25; α = 0.798, good)
I received sufficient medical knowledge concerning my child’s heart defect. I received the proper amount of medical information. I am convinced the physician’s explanation included all necessary details concerning my child’s condition. The possible consequences of my child´s treatment were adequately explained to me. Possible complications occurring during the treatment were explained well to me.
**2. Transparency regarding the Treatment Process** (sum-score range = 4 to 20; α = 0.808, very good)
After counseling, I knew what would be the next steps in my child’s treatment after delivery. It was explained to me in an understandable way when and in what order the following steps in my child’s treatment would take place. It was explained to me in an understandable way why the following steps in my child’s treatment would take place. During the conversation, my questions were adequately answered.
**3. Trust in Medical Staff** (sum-score range = 3 to 15; α = 0.811, very good)
Counseling has strengthened my trust in the medical institution. The conversation strengthened my trust in the physician. If possible, I would prefer that the same physician takes care of my baby after delivery.
**4. Perceived Situational Control** (sum-score range = 1 to 5, α not applicable as only one item)
During the conversation, I felt included in planning the treatment.
**5. Coping Resources** (sum-score range = 3 to 15, α = 0.743, good)
I felt treated with proper compassion. The conversation helped me to cope with my concerns and fears. During the conversation, my questions and concerns were taken seriously.

* Table 1 reused with permission from Georg Thieme Verlag KG, Klinische Pädiatrie, license number 5215890908463. Copyright stays with Georg Thieme Verlag KG, and any further reuse will need explicit permission from Georg Thieme Verlag KG.

**Table 2:** Multivariate Linear Regression Models—factors influencing “Overall Counseling Success” and the subdimensions of counseling success.

	Overall Counseling Success	Trust in Medical Staff	Transfer of Medical Knowledge	Coping Resources	Transparency Regarding the Treatment Process	Perceived Situational Control
Social Factors						
Interpersonal support by the physician during counseling	0.616 ***	0.670 ***	0.288 ***	0.567 ***	0.462 ***	0.317 ***
Frequent interruptions of the counseling by the physician	n.s.	n.s.	n.s.	n.s.	−0.223 ***	n.s.
Counseling in easy-to-understand terms	0.249 ***	n.s.	0.374 ***	0.160 **	0.129 *	n.s.
Spatiotemporal Factors						
Short period of time between suspected diagnosis and counseling	0.135 **	n.s.	n.s.	0.149 **	0.150 **	n.s.
No appropriate room during the consultation	n.s.	−0.119 *	n.s.	n.s.	n.s.	n.s.
Informational Factors						
Information materials received	n.s.	n.s.	n.s.	n.s.	n.s.	n.s.
Unfulfilled need to receive information material	n.s.	n.s.	−0.253 ***	n.s.	n.s.	n.s.
Information materials helped to answer upcoming questions independently	n.s.	n.s.	n.s.	n.s.	0.157 **	n.s.
Information how to obtain psychological support received	n.s.	n.s.	n.s.	n.s.	n.s.	n.s.
Control Variables						
Age	n.s.	n.s.	n.s.	n.s.	n.s.	0.166 *
First language German	n.s.	0.131 *	n.s.	n.s.	n.s.	n.s.
Gender	n.s.	n.s.	n.s.	n.s.	n.s.	n.s.
Sorrows	n.s.	n.s.	n.s.	n.s.	n.s.	n.s.
Permanent relationship	n.s.	n.s.	n.s.	n.s.	n.s.	n.s.
Social status	n.s.	n.s.	n.s.	n.s.	n.s.	n.s.
ISCED ^+^	n.s.	n.s.	n.s.	n.s.	n.s.	n.s.
Pre-existing medical knowledge	n.s.	n.s.	n.s.	n.s.	n.s.	n.s.
Severity of fetal CHD ^++^	n.s.	n.s.	n.s.	0.132 *	n.s.	n.s.
Location	0.102 *	0.112 *	n.s.	n.s.	0.103 *	n.s.
Counseling during COVID-19	n.s.	n.s.	n.s.	n.s.	n.s.	n.s.
Corrected R^2^	0.557	0.478	0.410	0.462	0.384	0.141
*n* ^+++^	191	208	213	206	187	218

The standardized regression coefficients are given (β-coefficients); * *p* < 0.05, ** *p* < 0.01, *** *p* < 0.001, n.s. = not significant. ^+^ ISCED: International Standard Classification of Education [27]. ^++^ Severity of diagnosed fetal CHD according to [7]. ^+++^ valid *n* for each item, as not all respondents answered all items completely (i.e., missing values were not replaced by mean values).

## Data Availability

Further data that support the findings of this study are available upon reasonable request from the corresponding author. Some data are not publicly available due to privacy or ethical restrictions.

## Appendix A

**Table A1:** Sample Structure (*n* = 226).

Variable	Expression	Valid % *
Gender	Female	59.3
	Male	40.7
Age	Mean value (years)	34.63
	Median (years)	35
	SD	5.404
Permanent relationship	Yes	90.3
	No	9.7
ISCED **	Low	6.2
	Medium	38.2
	High	55.6
Social status ***	Low	27.6
	Medium	43.3
	High	29.0
German first language	Yes	83.2
	No	16.8
Preexisting medical knowledge	Yes	31.1
	No	68.9
Sorrows ****	Major sorrows	35.8
	Intermediate sorrows	25.0
	Low sorrows	39.3
Location	Location A: centers 1 + 2	35.8
	Location B: centers 3 + 4	64.2
Counseling during the COVID-19-pandemic	Yes	25.2
	No	74.8
Gestational age at fetal cardiac diagnosis	Mean value (weeks + days)	24 + 1
	Median (weeks + days)	23
	Minimum (weeks + days)	9
	Maximum (weeks + days)	38
	SD (weeks + days)	6+1
Severity of fetal CHD *****	Low	11.6
	Medium	31.3
	High	57.1

* Differences to 100% are due to rounding. ** ISCED: International Standard Classification of Education; social status according to ISCED [27]. *** Social status according to occupation. **** “Sorrows” is defined by three items: “I am extremely concerned”, “I am unsure how to evaluate the situation”, “I think the situation is serious”. Internal consistency of this subscale was very good with a Cronbach’s α coefficient of 0.949. For further explanation see also [25]. ***** Severity of diagnosed fetal CHD according to [7].

**Table A2:** Summary of fetal cardiac diagnoses and extracardiac anomalies.

Fetal Cardiac Diagnosis	Genetic or Extra-Cardiac Findings	Number of Cases
AVSD	Trisomy 21	6
AVSD		5
AVSD, ARSA	Trisomy 21	1
AVSD, hypoplastic aortic arch, coarctation		1
AVSD, TOF	Trisomy 21	1
Coarctation		5
Coarctation	Turner syndrome	1
Coarctation, ARSA		1
Suspicion for coarctation, ventricular disproportion		2
Coarctation, aortic stenosis		1
Critical aortic stenosis		2
Critical aortic stenosis, severe mitral regurgitation, coarctation		1
Aortic stenosis, aortic arch hypoplasia, Perimembranous VSD		1
Borderline left ventricle, hypoplastic aortic arch, coarctation, LSVC		1
Critical pulmonary stenosis, severe tricuspid regurgitation		1
Severe pulmonary stenosis, severe tricuspid regurgitation		1
DILV, MGA, aortic arch hypoplasia		1
DILV, MGA, aortic arch hypoplasia, bilateral SVC		1
DORV		4
DORV, aortic arch hypoplasia, coarctation		1
DORV, MGA, right aortic arch, hypoplastic aortic arch, coarctation		1
DORV, subpulmonary stenosis		1
DORV, TGA		1
DORV, TGA, PA		1
DORV, TGA, subpulmonary stenosis		1
DORV, TOF type, right aortic arch, MAPCA		1
Severe Ebstein´s anomaly of the tricuspid valve		1
Ebstein´s anomaly of the tricuspid valve		2
Heterotaxy syndrome, AVSD, absent right AV connection, pulmonary stenosis, MGA, bilateral SVCs	Situs inversus abdominalis, asplenia	1
Heterotaxy syndrome, AVSD, pulmonary stenosis, MGA, right aortic arch		1
Heterotaxy syndrome, dextrocardia, DORV, pulmonary stenosis, MGA		1
Heterotaxy syndrome, HLHS, TAPVR, azygos continuation		1
HLHS		8
HLHS, DORV		1
HLHS, VSD		1
Hypoplastic aortic arch		2
Hypoplastic aortic arch, borderline LV		1
Hypoplastic aortic arch, coarctation		2
Hypoplastic aortic arch, perimembranous VSD		1
Hypoplastic aortic arch, VSD	* MCAD	1
Hypoplastic aortic arch, VSD muscular, PAPVR		1
IAA, borderline left ventricle	DiGeorge-syndrome	1
IAA, VSD		1
LAI, dextrocardia, hypoplastic right ventricle, tricuspid atresia, pulmonary stenosis, VSD, MGA		1
LSVC		1
LSVC, * ASD	Trisomy 21	1
* ASD	Trisomy 21	1
Mild tricuspid regurgitation		1
Non-compaction cardiomyopathy		1
PA, IVS		2
PA, IVS, sinusoids		1
PA, VSD		4
PA, VSD, MAPCAs		1
PA, VSD, TGA		1
PA/IVS, bipartite right ventricle, right ventricular hypertrophy		1
Pulmonary stenosis		1
Right aortic arch		1
TGA (complex)		10
TGA (simple)		8
ccTGA		2
TOF	Trisomy 21	1
TOF		6
TOF, right aortic arch	DiGeorge-syndrome	1
Tricuspid atresia		2
Tricuspid atresia Ib		1
Tricuspid valve dysplasia, moderate regurgitation, mild pulmonary stenosis, LSVC	Trisomy 21	1
Tricuspid valve dysplasia, prenatal duct closure		1
VSD	renal agenesis	1
VSD		4
VSD	* Reciprocal translocation chromosome 1 and 7; deletions: 1q43 and 7p15.3–p21.1	1
VSD	* Cystic fibrosis	1
VSD, hypoplastic aortic arch, coarctation, LSVC		1
VSD, inlet		1
VSD, muscular		1
VSD, perimembranous		1

* postnatal diagnosis. Abbreviations: ARSA aberrant right subclavian artery, AV atrioventricular, ASD atrial septal defect, AVSD atrioventricular septal defect, CC congenitally corrected, DORV double outlet right ventricle, HLHS hypoplastic left heart syndrome, IAA interrupted aortic arch, IVS intact ventricular septum, LAI left atrial isomerism, LSVC left superior vena cava, MAPCAs main aortopulmonary collateral arteries, MCAD medium-chain acyl-CoA dehydrogenase deficiency, MGA malposition of the great arteries, PA pulmonary atresia, PAPVR/TAPVR partial or total anomalous pulmonary venous return, TGA transposition of the great arteries, TOF tetralogy of Fallot, VSD ventricular septal defect.

**Table A3:** Overall counseling success and counseling success in the analytical subdimensions.

	Counseling Success:		
	Successful *	Satisfying *	Unsuccessful *
(a) Overall counseling success:	47.5%	52%	0.5%
(b) Subdimensions:			
Transfer of medical knowledge	49.1%	49.5%	1.4%
Trust in medical staff	72.8%	24.4%	2.8%
Transparency regarding the treatment process	63.8%	35.3%	0.9%
Coping resources	50.5%	45.0%	4.6%
Perceived situational control	45.7%	32.6%	21.7%

* The raw sum score (values of the corresponding Likert-scaled items summed to one score) was categorized into “successful” (values 1 and 2 of the underlying Likert-scaled items and their respective multiples), “satisfying” (value 3 of the underlying Likert-scaled items and their respective multiples), and “unsuccessful” (values 4 and 5 of the underlying Likert-scale items and their respective multiples).

## References

[1] Van Der Linde D., Konings E.E., Slager M.A., Witsenburg M., Helbing W.A., Takkenberg J.J., Roos-Hesselink J.W. (2011). Birth prevalence of congenital heart disease worldwide: A systematic review and meta-analysis. J. Am. Coll. Cardiol..

[2] Bonnet D., Coltri A., Butera G., Fermont L., Le Bidois J., Kachaner J., Sidi D. (1999). Detection of Transposition of the Great Arteries in Fetuses Reduces Neonatal Morbidity and Mortality. Circulation.

[3] Tworetzky W., McElhinney D.B., Reddy V.M., Brook M.M., Hanley F.L., Silverman N.H. (2001). Improved Surgical Outcome After Fetal Diagnosis of Hypoplastic Left Heart Syndrome. Circulation.

[4] Franklin O., Burch M., Manning N., Sleeman K., Gould S., Archer N. (2002). Prenatal diagnosis of coarctation of the aorta improves survival and reduces morbidity. Heart.

[5] Holland B.J., Myers J.A., Woods C.R. (2015). Prenatal diagnosis of critical congenital heart disease reduces risk of death from cardiovascular compromise prior to planned neonatal cardiac surgery: A meta-analysis. Ultrasound Obstet. Gynecol..

[6] Donofrio M.T., Moon-Grady A.J., Hornberger L.K., Copel J.A., Sklansky M.S., Abuhamad A., Cuneo B.F., Huhta J.C., Jonas R.A., Krishnan A. (2014). Diagnosis and treatment of fetal cardiac disease: A scientific statement from the American Heart Association. Circulation.

[7] Allan L.D., Huggon I.C. (2004). Counselling following a diagnosis of congenital heart disease. Prenat. Diagn..

[8] Allan L., Dangel J., Fesslova V., Marek J., Mellander M., Oberhänsli I., Oberhoffer R., Sharland G., Simpson J., Sonesson S.E. (2004). Recommendations for the practice of fetal cardiology in Europe. Association for European Paediatric Cardiology. Cardiol. Young.

[9] Arya B., Glickstein J.S., Levasseur S.M., Williams I.A. (2013). Parents of Children with Congenital Heart Disease Prefer More Information than Cardiologists Provide. Congenit. Heart Dis..

[10] Bratt E.L., Järvholm S., Ekman-Joelsson B.M., Mattson L.Å., Mellander M. (2015). Parent’s experiences of counselling and their need for support following a prenatal diagnosis of congenital heart disease—A qualitative study in a Swedish context. BMC Pregnancy Childbirth.

[11] Carlsson T., Bergman G., Wadensten B., Mattsson E. (2016). Experiences of informational needs and received information following a prenatal diagnosis of congenital heart defect. Prenat. Diagn..

[12] Rychik J., Donaghue D.D., Levy S., Fajardo C., Combs J., Zhang X., Szwast A., Diamond G.S. (2013). Maternal Psychological Stress after Prenatal Diagnosis of Congenital Heart Disease. J. Pediatr..

[13] Sklansky M., Tang A., Levy D., Grossfeld P., Kashani I., Shaughnessy R., Rothman A. (2002). Maternal psychological impact of fetal echocardiography. J. Am. Soc. Echocardiogr..

[14] Rosenberg K.B., Monk C., Glickstein J.S., Levasseur S.M., Simpson L.L., Kleinman C.S., Williams I.A., Simpson L.L. (2010). Referral for fetal echocardiography is associated with increased maternal anxiety. J. Psychosom. Obstet. Gynecol..

[15] Field T., Diego M., Hernandez-Reif M., Schanberg S., Kuhn C., Yando R., Bendell D. (2003). Pregnancy anxiety and comorbid depression and anger: Effects on the fetus and neonate. Depress. Anxiety.

[16] Field T. (2011). Prenatal depression effects on early development: A review. Infant Behav. Dev..

[17] Mulder E.J.H., de Medina P.G.R., Huizink A.C., Bergh B.R.H.V.D., Buitelaar J.K., Visser G.H.A. (2002). Prenatal maternal stress: Effects on pregnancy and the (unborn) child. Early Hum. Dev..

[18] Weinstock M. (2005). The potential influence of maternal stress hormones on development and mental health of the offspring. Brain, Behav. Immun..

[19] Talge N.M., Neal C., Glover V. (2007). Early Stress, Translational Research and Prevention Science Network: Fetal and neonatal experience on child and adolescent mental health. Antenatal maternal stress and long-term effects on child neurodevelopment: How and why?. J. Child Psychol. Psychiatry.

[20] Huizink A.C., De Medina P.G.R., Mulder E., Visser G.H., Buitelaar J.K. (2003). Stress during pregnancy is associated with developmental outcome in infancy. J. Child Psychol. Psychiatry.

[21] Kovacevic A., Bär S., Starystach S., Simmelbauer A., Elsässer M., Müller A., Mohammadi Motlagh A., Oberhoffer-Fritz R., Ostermayer E., Ewert P. (2020). Objective Assessment of Counselling for Fetal Heart Defects: An Interdisciplinary Multicenter Study. J. Clin. Med..

[22] Kovacevic A., Simmelbauer A., Starystach S., Elsässer M., Müller A., Bär S., Gorenflo M. (2020). Counseling for Prenatal Congenital Heart Disease—Recommendations Based on Empirical Assessment of Counseling Success. Front. Pediatr..

[23] World Health Organization Regional Office for Europe (WHO Europe) Health Literacy. The Solid Facts. https://apps.who.int/iris/bitstream/handle/10665/128703/e96854.pdf.

[24] Sørensen K., Van den Broucke S., Fullam J., Doyle G., Pelikan J., Slonska Z., Brand H., (HLS-EU) Consortium Health Literacy Project European (2012). Health literacy and public health: A systematic review and integration of definitions and models. BMC Public Health.

[25] Kovacevic A., Simmelbauer A., Starystach S., Elsässer M., Sohn C., Müller A., Bär S., Gorenflo M. (2018). Assessment of Needs for Counseling After Prenatal Diagnosis of Congenital Heart Disease—A Multidisciplinary Approach. Klinische Pädiatrie.

[26] Wolf C., Best H. (2010). Handbuch der Sozialwissenschaftlichen Datenanalyse.

[27] OECD (1999). Classifying Educational Programmes. http://www.oecd.org/dataoecd/41/42/1841854.pdf.

[28] Kovacevic A., Bär S., Starystach S., Elsässer M., van der Locht T., Mohammadi Motlagh A., Ostermayer E., Oberhoffer-Fritz R., Ewert P., Gorenflo M. (2021). Fetal Cardiac Services during the COVID-19 Pandemic: How Does It Affect Parental Counseling?. J. Clin. Med..

[29] Kovacevic A., Elsässer M., Fluhr H., Müller A., Starystach S., Bär S., Gorenflo M. (2021). Counseling for fetal heart disease—current standards and best practice. Transl. Pediatr..

[30] Bousquet G., Orri M., Winterman S., Brugière C., Verneuil L., Revah-Levy A. (2015). Breaking Bad News in Oncology: A Metasynthesis. J. Clin. Oncol..

[31] Storstein A. (2011). Communication and neurology—bad news and how to break them. Acta Neurol. Scand..

